# Impact of Host Resistance to Tomato Spotted Wilt Orthotospovirus in Peanut Cultivars on Virus Population Genetics and Thrips Fitness

**DOI:** 10.3390/pathogens10111418

**Published:** 2021-11-01

**Authors:** Pin-Chu Lai, Mark R. Abney, Sudeep Bag, Albert K. Culbreath, Rajagopalbabu Srinivasan

**Affiliations:** 1 Department of Entomology, University of Georgia, Griffin, GA 30223, USA; pclai@uga.edu; 2 Department of Entomology, University of Georgia, Tifton, GA 31793, USA; mrabney@uga.edu; 3 Department of Plant Pathology, University of Georgia, Tifton, GA 31793, USA; Sudeepbag@uga.edu (S.B.); spotwilt@uga.edu (A.K.C.)

**Keywords:** host plant resistance, tolerance, *Orthotospovirus*, spotted wilt of peanut, *Frankliniella fusca*, vector fitness

## Abstract

Thrips-transmitted tomato spotted wilt orthotospovirus (TSWV) is a major constraint to peanut production in the southeastern United States. Peanut cultivars with resistance to TSWV have been widely used for over twenty years. Intensive usage of resistant cultivars has raised concerns about possible selection pressure against TSWV and a likelihood of resistance breakdown. Population genetics of TSWV isolates collected from cultivars with varying levels of TSWV resistance was investigated using five TSWV genes. Phylogenetic trees of genes did not indicate host resistance-based clustering of TSWV isolates. Genetic variation in TSWV isolates and neutrality tests suggested recent population expansion. Mutation and purifying selection seem to be the major forces driving TSWV evolution. Positive selection was found in N and RdRp genes but was not influenced by TSWV resistance. Population differentiation occurred between isolates collected from 1998 and 2010 and from 2016 to 2019 but not between isolates from susceptible and resistant cultivars. Evaluated TSWV-resistant cultivars differed, albeit not substantially, in their susceptibility to thrips. Thrips oviposition was reduced, and development was delayed in some cultivars. Overall, no evidence was found to support exertion of selection pressure on TSWV by host resistance in peanut cultivars, and some cultivars differentially affected thrips fitness than others.

## 1. Introduction

Resistant cultivars often form the first line of defense against arthropod-borne plant viruses such as the thrips-transmitted tomato spotted wilt orthotospovirus (TSWV). TSWV causes substantial economic losses in various crops such as tomato, pepper, tobacco, and peanut [1,2,3,4,5,6]. TSWV infection leads to spotted wilt disease in peanut, which has been a severe limiting factor in peanut (*Arachis hypogaea* L.) production in the southeastern United States. TSWV infection in peanut was first reported in Texas in 1971 and has since spread to other southern states [2,7].

In the 1990s, TSWV became a yield-limiting problem in peanut in the southeastern United States [2,8,9,10]. Observations then indicated variation in susceptibility to TSWV among peanut cultivars. For example, the commonly grown cultivar ‘Florunner’ was found to be highly susceptible to TSWV, while another cultivar ‘Southern Runner’ was less susceptible to TSWV [11,12]. Intensive screening and breeding efforts over the next three decades led to consistent releases of peanut cultivars with incremental levels of TSWV resistance [6]. Resistance to TSWV in peanut is commonly referred to as field resistance or tolerance and is typified by milder symptoms following TSWV infection and increased yield compared with TSWV-susceptible cultivars, especially under high virus pressure [2,13]. The mode of TSWV resistance in peanut is not completely known and is different from crops such as tomato and pepper. In pepper and tomato, dominant genes such as *Tsw* and *Sw-5* confer resistance via hypersensitive response (HR) characterized by rapid death of cells around virus entry sites, causing local necrotic lesions without systemic symptoms [14,15]. However, HR was not observed in TSWV-resistant peanut cultivars; instead, TSWV infection in field-resistant peanut cultivars resulted in systemic symptom expression, albeit to a lesser degree than in susceptible cultivars [2,6,16]. These responses suggested that field resistance is more likely to be governed by multiple quantitative traits in peanut as opposed to single gene-governed resistance in Solanaceae hosts. Several major quantitative trait loci (QTLs) have thus far been linked to TSWV resistance in peanut cultivars [17,18,19,20]. Yet, the mechanism of TSWV resistance in peanut remains to be characterized.

TSWV strains have overcome resistance conferred by single genes such as *Tsw* in pepper and *Sw-5* in tomato in several places worldwide [14,21,22,23,24]. Resistance-breaking (RB) TSWV strains originated from mutations leading to positive selection and/or reassortment [25,26,27,28]. While TSWV RB strains have not been reported in peanut, the potential threat of emergence of RB strains remains a concern. TSWV management in peanut relies heavily on resistant cultivars with an estimated >95% of the peanut acreage planted with them [6]. As stated earlier, these field-resistant peanut cultivars display less severe symptoms and accumulate less TSWV following TSWV infection than susceptible cultivars [16]. Whether the prolonged resistant cultivar–TSWV interactions in the peanut production landscape could lead to development of new strains that can overcome field resistance remains to be assessed.

TSWV has a tripartite genome consisting of large (8.9 kb), medium (4.8 kb), and small (2.9 kb) segments with coding regions for five genes [29]. The large segment encodes for the RNA-dependent RNA polymerase (RdRp), which plays a crucial role in genome replication [30,31]. The medium segment encodes for a non-structural protein (NSm) in the positive sense and the Gn/Gc glycoprotein precursor in the negative sense [32]. NSm is involved in cell-to-cell movements in plant hosts [33,34,35]. The Gn/Gc glycoproteins play a role in maturation and assembly of virions as well as thrips transmission [29,36]. The small segment encodes for another nonstructural protein (NSs) in the positive sense and the nucleocapsid protein (N) in the negative sense [37]. NSs was identified as RNA silencing suppressor during plant infection [38], and the N protein encapsidates the RNA segments to form ribonucleoprotein [39]. Heterogeneity in nucleotide sequences of NSs and NSm between wild type and RB strains of TSWV leading to positive selection was associated with resistance breakdown in pepper and tomato [25,26,28]. Sundaraj et al. (2014) [40] did not find evidence of positive selection pressure on the N gene of TSWV isolates collected from TSWV-resistant peanut cultivars. However, the effect of possible selection pressure from resistance in peanut cultivars on other genes in the TSWV genome remained unexamined.

TSWV is exclusively transmitted by thrips in a persistent and propagative mode under natural conditions, and resistance or tolerance against the vector could also influence the susceptibility of these cultivars to TSWV [41,42,43,44,45]. Resistance to thrips could impact thrips preference, feeding, reproduction, and development and could ultimately affect virus acquisition and inoculation [16,40,45]. Therefore, it is possible that the observed field resistance to TSWV could be due to effects against the virus and/or the vector.

In this study, TSWV isolates collected from peanut cultivars with varying levels of TSWV field resistance were studied by fully or partially sequencing the five TSWV genes and assessing numerous population genetics parameters. In addition, whether peanut cultivars possessed any resistance/tolerance to thrips and if that interfered with TSWV transmission was investigated.

## 2. Results

### 2.1. Phylogeny of TSWV Isolates

**Figure 1:**
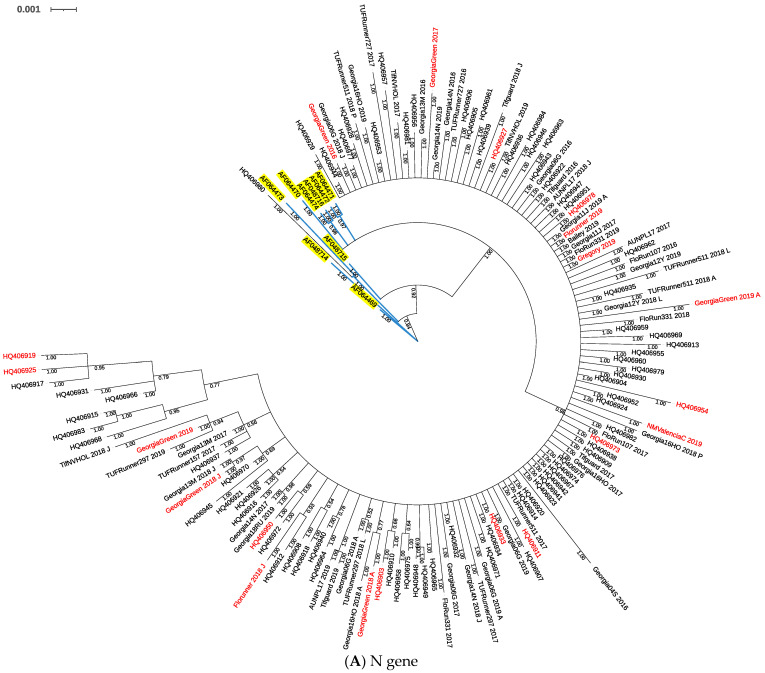

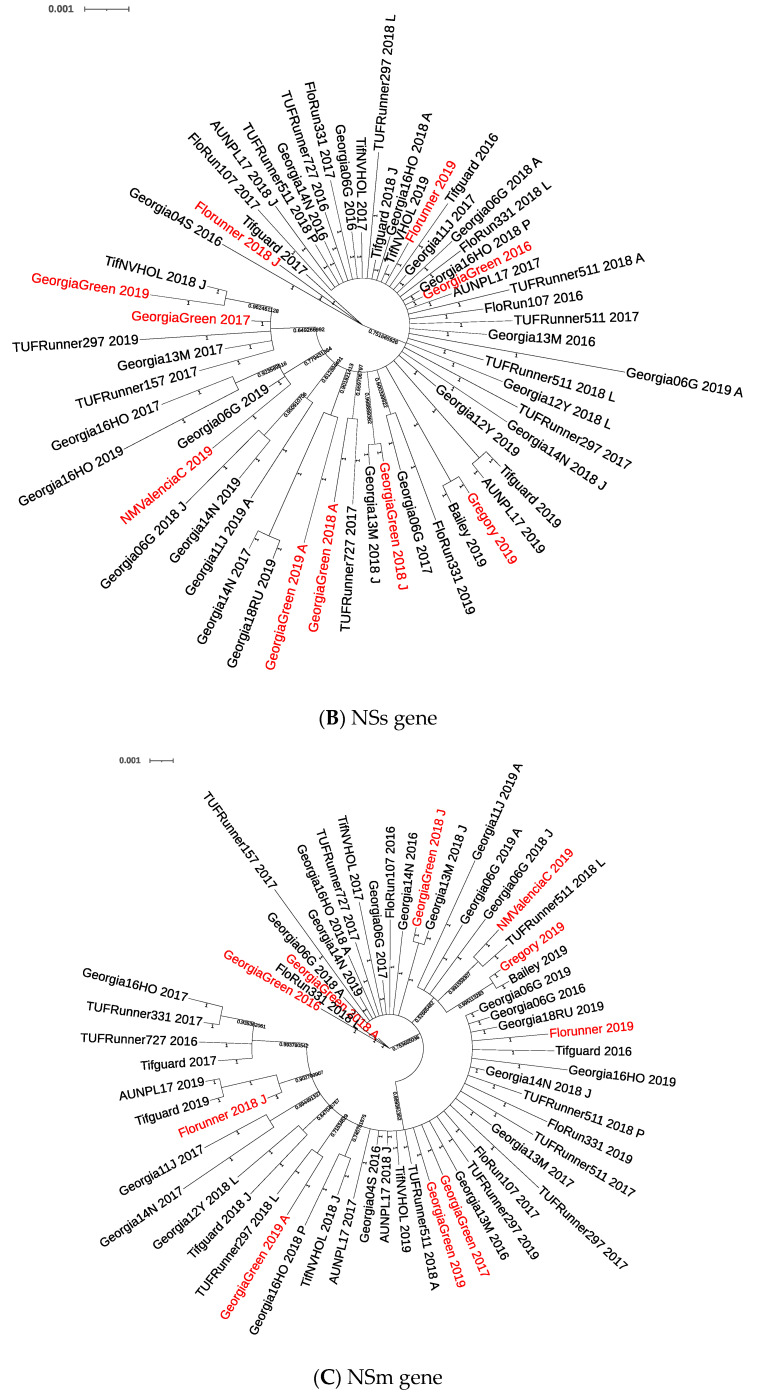

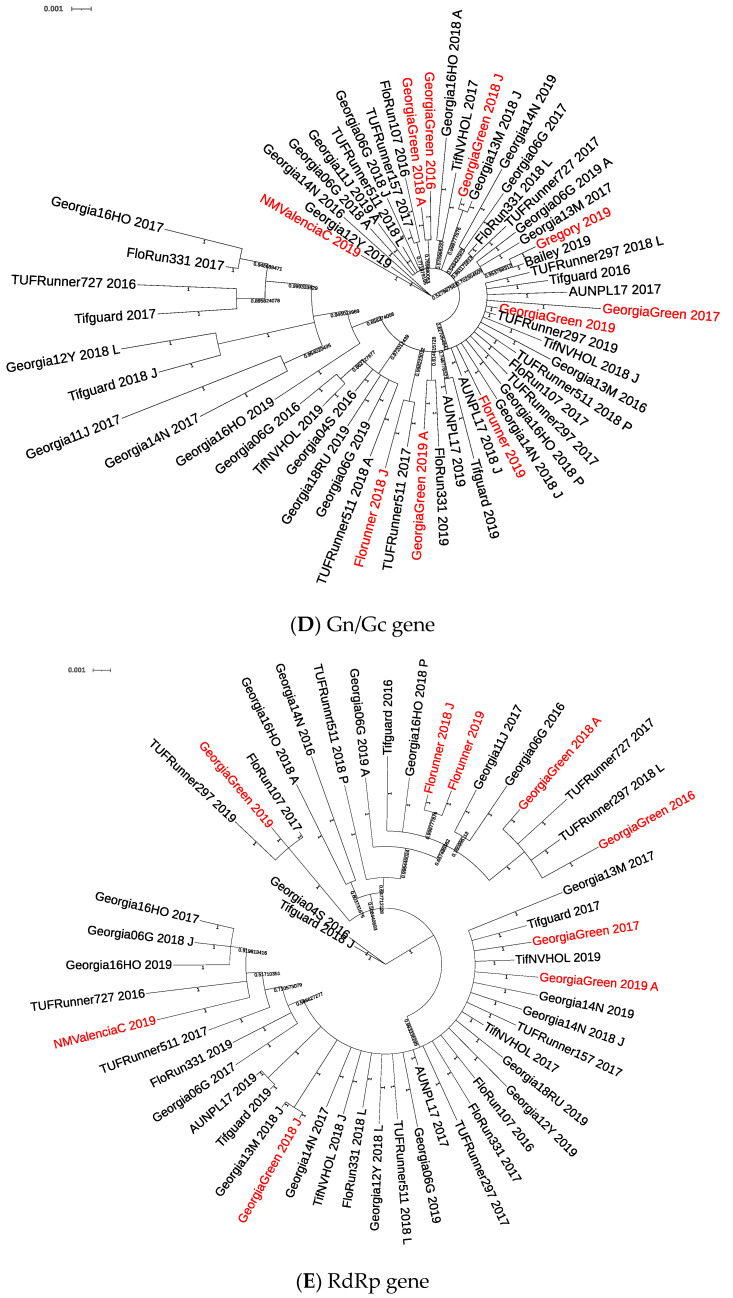
Phylogenetic tree of TSWV isolates from TSWV-susceptible and resistant peanut cultivars based on Bayesian analysis of TSWV N (**A**), NSs (**B**), NSm (**C**), Gn/Gc (**D**), and RdRp (**E**) nucleotide sequences. Analysis was conducted using MrBayes v3.2.7, and the phylogenetic tree was constructed using Interactive Tree of Life. Whole gene sequences were used for the N gene, and partial sequences were used for all others. Posterior probability values for each branch are labeled. Sample IDs in red are isolates collected from TSWV-susceptible peanut cultivars. In the N gene phylogenetic tree (**A**), sample IDs with accession numbers starting with “AF” and highlighted in yellow were collected in 1998 [46], and accession numbers starting with “HQ” were collected in 2010 [40].

Phylogenetic trees were constructed based on nucleotide polymorphisms observed in full length of the N gene, and partial sequences of NSs, NSm, Gn/Gc, and RdRp genes using Bayesian inferences. The N gene phylogenetic tree consisted of 150 sequences including isolates from 1998, 2010, and 2016–2019 (Figure 1A). The nine isolates collected from 1998 were grouped in a small clade closer to the base of the tree apart from isolates collected in 2010 and 2016–2019, except one isolate collected in 2010 grouped within a clade with isolates from 1998. Isolates collected in 2010 and 2016–2019 blended in a big clade of the tree. Isolates collected from susceptible cultivars in 2016–2019 were dispersed within the clade without obvious distinction with isolates from resistant cultivars (Figure 1A).

Phylogenetic trees of NSs (Figure 1B), NSm (Figure 1C), Gn/Gc (Figure 1D), and RdRp (Figure 1E) included several clades; however, nucleotide polymorphisms in TSWV isolates were not associated with host susceptibility to TSWV. 

### 2.2. Genetic Diversity, Test of Neutrality, and Identification of Selection

Across all the TSWV genes, low nucleotide diversity was observed ranging from 0.0072 to 0.0132 (Table 1). Mutations were observed in all five genes as indicated by detection of segregating sites, and the number of segregating sites was proportional to the sample size (Table 1). Genetic diversity was compared among TSWV isolates collected from 1998, 2010, and 2016–2019 using N gene sequences. TSWV isolates collected in 1998 had slightly higher nucleotide diversity than isolates collected in 2010 and new isolates collected in 2016 to 2019 (Table 1). Overall, the Gn/Gc gene had the highest population mutation rate (θ_w_) followed by the RdRp gene, while the NSs gene had the lowest (Table 1). Across genes, the resistant subgroup had higher θ_w_ than the susceptible subgroup. However, the population size of the resistant subgroup was larger than the susceptible subgroup.

For all population subgroups across five genes, negative values of Tajima’s D and Fu and Li’s D * and F * statistics were found, and most of the statistics were significant except for a few subgroups with a smaller sample size (Table 1). Negative values of the statistics Tajima’s D and Fu and Li’s D * and F * tests suggested recent population expansion events or populations under purifying selection.

Selection tests found eight codon positions of the N gene sequence with overabundance of non-synonymous substitutions indicated by positive values of posterior β-α (β: posterior non-synonymous substitution rate; α: posterior synonymous substitution rate), while only one codon at position 7 (T→I, F) was determined to be significantly driven by positive selection (Table 2). Among 258 codons of the N gene sequence, negative selection (i.e., purifying selection) was found at 63 sites (24%). Selection tests showed that nine, two, and six codon positions had an excess of non-synonymous substitutions in NSs, NSm, and Gn/Gc sequences, respectively; however, none were significant, and no positive selection was determined in those three genes (Table 2). Among 286, 224, and 251 codons of the NSs, NSm, and Gn/Gc sequences, significant negative selection was observed at 17 (6%), 29 (13%), and 47 (18%) codon sites, respectively. Selection tests found 12 codon positions of RdRp sequence with overabundance of non-synonymous substitutions, and positive selection was determined at codon position 149 (K→R), 207 (Q→R), 368 (G→R, E, A), and 397 (Q→L, H). Among 471 codons of the RdRp sequence, 110 (23%) codon positions were found with evidence of negative (purifying) selection (Table 2). Within N and RdRp sequences with significant non-synonymous substitutions, non-synonymous substitutions were found in a subset of TSWV isolates from both susceptible and resistant cultivars, which indicated the lack of association between cultivar susceptibility to TSWV and non-synonymous substitutions.

### 2.3. Genetic Differentiation between Subgroups of TSWV Isolates

According to the nucleotide-based population differentiation statistics (Ks and Kst, and Snn) on N gene sequences, significant genetic differentiation was observed between the subgroups of isolates collected in 1998, 2010, and in this study from 2016 to 2019 (Table 3). The extent of differentiation was higher between 1998 isolates and the later subgroups of isolates (2010, 2016–2019, and after 2010) than between 2010 isolates and new isolates collected from 2016 to 2019 indicated by the Fst value, which was 10 times higher in the former than the later subgroup comparisons (Table 3). Except for the significant Snn statistic found between susceptible and resistant subgroups from 2010 isolates with the N gene, population differentiation was not found in the rest of the comparisons between susceptible and resistant subgroups, as none of the differentiation test statistics were significant across the five genes (Table 3). Small Fst values of the comparisons between susceptible and resistant subgroups indicated that nucleotide differences were similar between pairwise sequences from within and between subpopulations with no evidence of population differentiation. 

### 2.4. Thrips Feeding Injury and Survival

**Figure 2:**
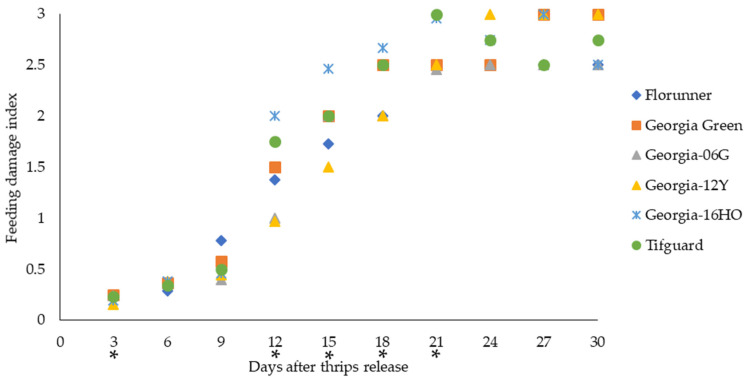
Thrips feeding injury evaluated at 3-day intervals for up to 30 days after thrips released on TSWV-susceptible and -resistant peanut cultivars. Florunner and Georgia Green are TSWV-susceptible cultivars, and Georgia-06G, Georgia-12Y, Georgia-16HO, and Tifguard are TSWV-resistant cultivars. Median feeding damage indices are presented for each evaluating day. Evaluating days denoted with “*” indicate significant cultivar effects on thrips feeding injury at *p* < 0.05 based on the Wilcoxon Score and Kruskal–Wallis test. Six plants of each cultivar were placed in a thrips-proof cage, and ten thrips were released at the base of each plant at the two-node stage. The experiment was conducted two times (*n* = 12 for each cultivar).

Two TSWV-susceptible cultivars, Florunner and Georgia Green, and four TSWV-resistant cultivars, Georgia-06G, Georgia-12Y, Georgia-16HO, and Tifguard, were examined for their susceptibility to thrips. Adult thrips were released onto peanut plants at the two-node stage, and thrips feeding injury on peanut leaves, presented as feeding damage index, was monitored from 3 to 30 days after thrips release (DAT). Thrips feeding injury varied with peanut cultivar at 3 (*χ*^2^ = 11.73, *p* = 0.0385), 12 (*χ*^2^ = 25.86, *p* < 0.0001), 15 (*χ*^2^ = 19.29, *p* = 0.0017), 18 (*χ*^2^ = 24.09, *p* = 0.0002), and 21 (*χ*^2^ = 13.66, *p* = 0.0179) DAT. At 3 DAT, Georgia-12Y had lower thrips feeding injury than Georgia-06G and Georgia Green based on the Wilcoxon-score; Georgia-12Y had the lowest median FDI, which was 1.6 times lower than the highest median FDI of Georgia Green (Figure 2). At 12 DAT, Georgia-12Y and Georgia-06G had lower thrips feeding injury than Georgia-16HO and Georgia Green; Georgia-12Y also had lower thrips feeding injury than Tifguard, and Florunner had lower thrips feeding injury than Georgia-16HO based on the Wilcoxon-score. Georgia-12Y had the lowest median FDI, which was 2.1 times lower than the highest median FDI of Georgia-16HO (Figure 2). At 15 DAT, Georgia-12Y and Georgia-06G had lower thrips feeding injury than Georgia-16HO, Georgia Green, and Tifguard, while Florunner had lower thrips feeding injury than Georgia Green based on the Wilcoxon-score. Georgia-12Y had the lowest median FDI, which was 1.6 times lower than the highest median FDI of Georgia-16HO (Figure 2). At 18 DAT, Georgia-12Y, Georgia-06G, and Florunner had lower thrips feeding injury than Georgia-16HO, Georgia Green, and Tifguard based on the Wilcoxon-score. Georgia-12Y, Georgia-06G, and Florunner all had lower median FDIs, which were at least 1.3 times lower than the highest median FDI of Georgia-16HO (Figure 2). At 21 DAT, Tifguard had higher thrips feeding injury than Georgia-12Y, Georgia-06G, Georgia Green, and Florunner, while Georgia-16HO had higher thrips feeding injury than Georgia-06G based on the Wilcoxon-score. Georgia-06G had the lowest median FDI, which was 1.2 times lower than the highest median FDI of Tifguard (Figure 2).

**Figure 3:**
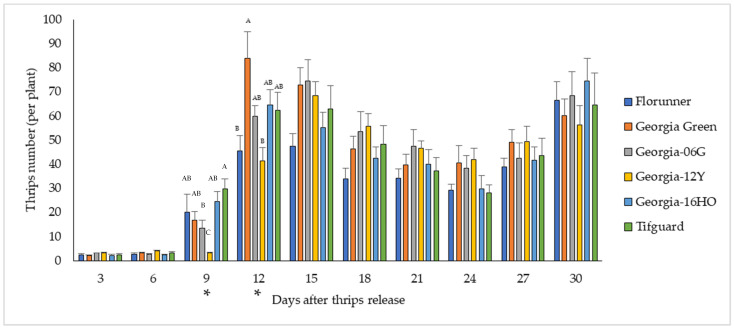
Mean number of thrips surviving on peanut plants over the course of 3–30 days after thrips released on TSWV-susceptible and -resistant cultivars. Florunner and Georgia Green are TSWV-susceptible cultivars, and Georgia-06G, Georgia-12Y, Georgia-16HO, and Tifguard are TSWV-resistant cultivars. Evaluation days denoted with “*” indicate significant cultivar effects on thrips survival, and bars labelled with the same letter were not significantly different from each other at *p* < 0.05. Six plants of each cultivar were placed in a thrips-proof cage, and ten thrips were released at the base of each plant at the two-node stage. The experiment was conducted two times (*n* = 12 for each cultivar).

Thrips survival was evaluated by counting all adult and immature thrips on each plant. The number of thrips surviving varied with peanut cultivar (F_5, 583_ = 2.52, *p* = 0.0286), and the variation among cultivars differed by recording time (F_45, 583_ = 3.09, *p* < 0.0001) (Figure 3, Supplementary Table S1). Significant differences in thrips survival among peanut cultivars were only observed at 9 and 12 DAT. At 9 DAT, the lowest number of thrips was found on Georgia-12Y, while at 12 DAT, fewer thrips were found on Georgia-12Y and Florunner than on other cultivars (Figure 3, Supplementary Table S1).

Overall, our results suggested that the susceptibility of peanut cultivars to *F. fusca* was similar with some minor variations in thrips feeding injury and survival during the experimental period.

### 2.5. Thrips Development, Reproduction, and Oviposition

**Figure 4:**
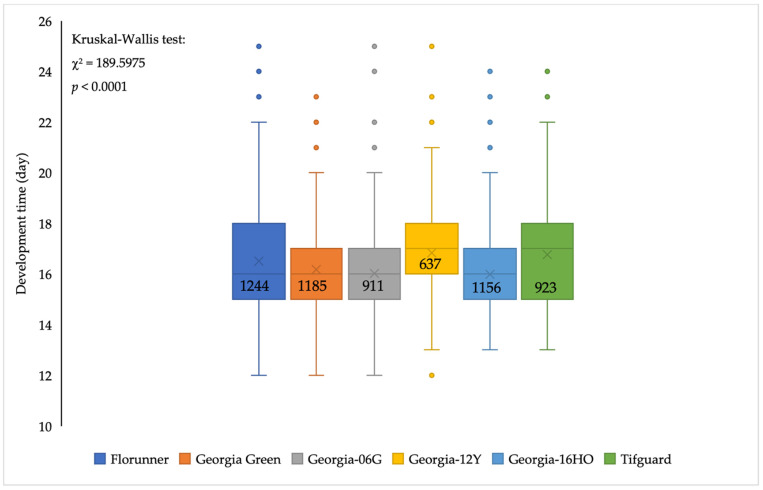
Developmental time of *Frankliniella fusca* to complete one generation (adult to adult) on leaflets of TSWV-susceptible and -resistant peanut cultivars. Florunner and Georgia Green are TSWV-susceptible cultivars, and Georgia-06G, Georgia-12Y, Georgia-16HO, and Tifguard are TSWV-resistant cultivars. The ‘horizontal line’ and ‘X’ in the box are median and mean developmental time, respectively. Dots are outliers based on 1.5 times of interquartile range. Sample size is labelled in the box. Ten female adult thrips were released on leaflets in each Munger cage and allowed to lay eggs for 72 h and removed; cages were monitored at 24 h intervals to record newly emerged adult thrips. Data were analyzed by Wilcoxon tests of NPAR1WAY procedure in SAS; significance of the test was determined by Kruskal–Wallis test.

**Figure 5:**
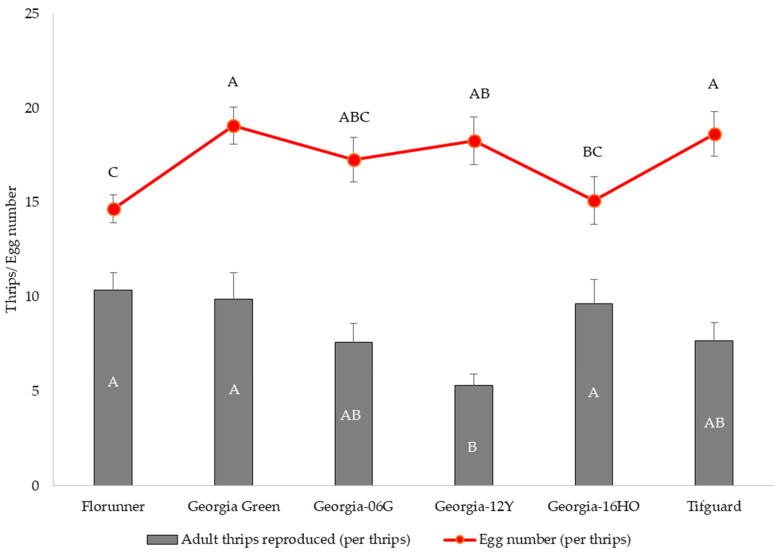
Mean and standard error of number of adult *Frankliniella fusca* produced and number of eggs oviposited per female released on leaflets of TSWV-susceptible and -resistant peanut cultivars. Florunner and Georgia Green are TSWV-susceptible cultivars, and Georgia-06G, Georgia-12Y, Georgia-16HO, and Tifguard are TSWV-resistant cultivars. Ten female adult thrips were released on leaflets in each Munger cage and allowed to lay eggs for 72 h and removed; cages were monitored at 24 h intervals to record newly emerged adult thrips. Mean thrips number followed by the same letter are not significantly different from each other at *p* < 0.05. Five female adult thrips were released on leaflets in each petri dish cage and allowed to lay eggs for 72 h and removed; eggs were stained and counted under a dissecting microscope.

The median developmental time for *F. fusca* to complete one generation varied among peanut cultivars (*p* < 0.05) with minor differences. The median developmental time of *F. fusca* on Georgia-12Y and Tifguard was one day longer than the rest of the cultivars (Figure 4). Thrips reproduction varied among peanut cultivars (F_5, 55_ = 5.10; *p* = 0.0007); the number of adult thrips emerging per one thrips released was significantly lower on Georgia-12Y than on Florunner, Georgia Green, and Georgia-16HO (Figure 5). Oviposition rate of thrips also varied among peanut cultivars (F_5, 55_ = 2.45; *p* = 0.0451). Number of eggs oviposited by thrips was higher in Georgia Green and Tifguard than Georgia-16HO and Florunner. In addition, oviposition rate was higher on Georgia-12Y than Florunner (Figure 5).

## 3. Discussion

Development of highly virulent isolates due to host resistance-induced selection pressure has led to the breakdown of host resistance to arthropod-borne viruses such as TSWV and has seriously impacted management [47,48]. The increase in TSWV incidence in recent years despite the intense use of resistant cultivars over twenty years in much of the acreage has created concerns about the stability of field resistance in peanut and possible changes in TSWV virulence [2,6,8,49,50,51,52]. An earlier study found no direct evidence to support the hypothesis that field resistance in peanut exerts selection pressure on TSWV, but that study was based on the evaluation of the TSWV N gene alone [40]. It is possible that alterations in other genes could also influence the virulence of isolates/strains [25,26,28]. The main objective of this study was to investigate if TSWV resistance in peanut exerts any selection pressure on any and/or all five genes of TSWV, namely N, NSs, Gn/Gc, NSm, and RdRp. In addition, this study examined the various population genetics factors shaping the local TSWV population structure, and whether the factors varied with virus genes.

According to phylogenetic analyses, nucleotide polymorphisms were observed in all five TSWV genes, but no distinct clustering of TSWV isolates based on host resistance in peanut cultivars was found in any of the genes evaluated. On the other hand, N gene sequences of TSWV isolates collected in 1998 parsed out from the sequences of isolates collected after that. The TSWV isolates collected from 2010 and 2016–2019 did not separate into different clades. These results demonstrated a significant temporal effect from 1998 to 2019 on TSWV populations in Georgia. These results should be cautiously interpreted as, except for N, all other genes were partially sequenced.

Overabundance of non-synonymous substitutions were found at codon positions in all five TSWV genes, while only one codon in N gene and four codons in RdRp were under positive selection. The non-synonymous amino acid substitutions were both conservative replacements and non-conservative—meaning that amino acid substitutions were not similar (different charge, polarity, and side chains). These changes did not seem to disrupt protein functions, as the virus interactions resulted in typical TSWV infection. Non-synonymous substitutions were found in both TSWV-susceptible and -resistant cultivars, which indicated that positive selection might not be related to TSWV susceptibility of the peanut hosts. On the contrary, significant negative selection (i.e., purifying selection) was found in up to 24% of the codons in the five TSWV genes. These results were similar to the findings in previous studies, where limited positive selection at codon sites was found across the five TSWV genes with the background of predominant purifying selection [40,53,54]. Among the five genes of TSWV, NSm had the fewest codon sites with overabundance of non-synonymous substitutions. Tsompana et al. (2005) also found that the NSm gene was the only gene in which no evidence of positive selection was found. NSm interacts with nucleocapsid protein and is associated with cellular membranes to facilitate cell-to-cell movement through plasmodesmata in host plants [33,34,35,55,56,57]. The NSm gene of TSWV was identified as an avirulence determinant of *Sw-5* gene-based resistance in tomato [58,59]. In addition, non-synonymous substitution of a codon in the NSm gene was found to be positively selected in TSWV resistance-breaking isolates against the *Sw-5* gene in tomato [26]. On the other hand, the TSWV NSs gene was identified as an avirulence determinant for *Tsw*-based resistance in pepper [60,61,62]. *Tsw*-based resistance in pepper was overcome by a single mutation in the NSs gene [25]. The NSs gene encodes a protein associated with RNA silencing suppression in infected plant hosts [38]. Nevertheless, positive selection was not found in the TSWV NSm or NSs gene in this study. In another study, positive selection was found in one codon site each of N, NSm, and RdRp genes of TSWV isolates collected from peanut in the mid-Atlantic states; those isolates with positive selection were collected in a certain year but not from other years, suggesting a possible strong environmental influence [54]. However, positive selection on N and RdRp genes was found in isolates from different collection years and locations in this study, suggesting that the source of selection pressure might appear commonly in local geographic regions over time. These results should be interpreted with the caveat that except for the N gene, all other genes were only partially sequenced, hence other non-synonymous substitutions occurring in non-sequenced areas of the genes could have been missed.

Low nucleotide diversity was found in all five TSWV genes; our results are consistent with sequences of the N gene from isolates collected in Georgia earlier [40], sequences of N, NSm, and RdRp from isolates collected in North Carolina and Virginia [54], as well as sequences of N, NSs, NSm, and Gn/Gc from isolates in different geographic regions worldwide [53]. The negative and significant neutrality test statistics, including Tajima’ D, and Fu and Li’s D * and F *, suggested that TSWV populations deviated from neutrality and likely experienced population expansion and/or purifying selection. The findings of this study aligned with previous studies on TSWV in which purifying selection and population expansion were also documented [40,53,54].

Temporal population differentiation between TSWV subgroups of isolates sampled during a 20-year period was evident by nucleotide-based statistics. However, no such effect was observed in any of the TSWV genes between subgroups of TSWV isolated from susceptible and resistant cultivars. These results echo previous findings and reaffirm the significance of the temporal effect on population genetic structure, leading to population differentiation of TSWV isolates collected from different years [40]. Altogether, this study reiterates previous findings that population expansion, purifying selection, and population differentiation are the major mechanisms shaping population genetics of TSWV [53,54].

Mutation, recombination, and reassortment are major mechanisms contributing to genetic variation in plant viruses [63,64]. All the mutations in TSWV genes identified in this study were from nucleotide substitutions and not from insertions or deletions (data not shown). Substitutions, indicated by number of segregating sites, were found in all TSWV genes, and the substitution rate varied among TSWV genes. The population mutation rate of each TSWV gene estimated in this study was similar or slightly lower than those reported from other geographic regions [53,54]. These results supported the previous finding that mutation is another significant evolutionary factor for shaping the population genetic structure of TSWV. Population mutation rate (θ_w_) also varied by subgroups of TSWV susceptibility of peanut cultivars. The mutation rate was higher in TSWV isolates collected from resistant cultivars than susceptible cultivars. However, these results need to be interpreted carefully, as θ_w_ might be affected by the sample size. Although recombination in negative-sense RNA viruses is much rarer, recombination in TSWV has been detected in TSWV genes and genomes in previous studies [54,65,66]. On the other hand, reassortment has also been found to play an important role in creating genetic variation in the TSWV genome [66]. TSWV has been shown to utilize reassortment to overcome TSWV N gene-derived resistance [67,68]. Since analyses were conducted on individual partial gene sequences but not with whole genome sequences in this study, recombination and reassortment were not tested in this study.

Alongside TSWV resistance, previous studies also have identified significant impacts of TSWV-resistant peanut cultivars on thrips biology and fitness [16,40]. In greenhouse no-choice tests conducted in this study, thrips feeding injury and survival was not different among peanut cultivars at the end of the experiment, when all the cultivars had high level of thrips densities and thrips feeding injuries, which suggested that none of the tested cultivars possessed a high level of resistance to *F. fusca*. Georgia-12Y had reduced *F. fusca* feeding injury and survival than other tested cultivars. In addition, a longer median developmental time of *F. fusca* to complete one generation and reduced reproduction on leaflets of Georgia-12Y both support the negative effect of Georgia-12Y on *F. fusca* development and fitness. In contrast, another study revealed that negative fitness effects of TSWV-resistant peanut cultivars on thrips resulted in reduced developmental time [16]. Developmental time was also reduced on peppers possessing resistance to thrips [69]. Divergent effects of host plant resistance on thrips developmental time could be related to the differences in resistance mechanisms. Thrips resistance in other crops was mediated via morphological traits of the plants such as leaf thickness, waxiness, and amount of pubescence; biochemical traits such as alkaloids and other secondary metabolites are also known to contribute to resistance to thrips [45]. Specific factors causing differences in susceptibility of peanut cultivars to thrips in this study are unknown and require further research.

The current study demonstrated that nucleotide substitutions were the important sources of genetic variation in TSWV. Population expansion and purifying selection were substantial factors driving TSWV evolution, while positive selection was occasionally found in N and RdRp genes. Overall, this study did not find evidence of TSWV resistance in peanut cultivars exerting substantial selection pressure on any of the five TSWV genes. Quantitative resistance is generally more durable than qualitative resistance (i.e., resistance conferred by a single resistance gene), likely because of the partial resistance effect exerting a low selection pressure on the pathogen. If the quantitative resistance is conferred by a combination of resistance mechanisms with multiple genes involved, the resistance would be more difficult to overcome [70,71]. The underlying mechanisms of TSWV resistance in peanut is just beginning to be revealed. A recent study suggested that defense-related genes and defense pathways that contribute resistance to viruses were upregulated in the resistant cultivar in comparison with the susceptible cultivar [72]. The roles of these genes in conferring resistance to TSWV are yet to be functionally validated. With indicators pointing to quantitative resistance against TSWV in peanut, monitoring for resistance-breaking strains in peanut might still be necessary, as even quantitative resistance could also be overcome by the pathogen [17,18,19,20,73]. TSWV-resistant peanut cultivars tested in this study mostly possess similar levels of susceptibility to *F. fusca* except in the case of Georgia-12Y. The negative impact of some TSWV-resistant peanut cultivars such as Georgia-12Y on thrips fitness could likely contribute to the overall success of TSWV resistance cultivars. Interaction between thrips vectors and peanut is a crucial part of the TSWV pathosystem, and thrips resistance in peanut cultivars in addition to TSWV resistance could provide enhanced and durable resistance.

## 4. Materials and Methods

### 4.1. TSWV Isolates

TSWV symptomatic leaves were collected from 22 peanut cultivars with varying levels of field resistance to TSWV during the growing season of 2016 to 2019 in Georgia. Foliage samples (*n* = 59) were collected from peanut fields on research farms at the University of Georgia Tifton campus and Attapulgus Research and Education Center in Georgia. Peanut type, cultivar, collection year and location, and TSWV susceptibility of each cultivar are listed in Table 4.

### 4.2. RNA Extraction, cDNA Synthesis, PCR, and Sequencing

Total RNA from symptomatic leaf samples of susceptible and resistant peanut cultivars was extracted using an RNeasy plant mini kit (Qiagen, Valencia, CA, USA) following the manufacturer’s protocol. Approximately 0.1 g of leaf tissue per sample was used for RNA extraction. The total RNA extract was used as the template for complementary DNA (cDNA) synthesis using a Go-Script reverse transcription system (Promega Corporation, Madison, WI, USA) with oligo dT primers following the manufacturer’s instructions. The synthesized cDNA served as the template for PCR. PCR was conducted in a DNA engine thermo cycler (Bio-Rad Laboratories, Hercules, CA, USA) using 50 μL volume reactions. Primers were designed to amplify the full length of the N gene and partial regions of NSs, NSm, Gn/Gc, and RdRp genes according to the reference sequences of the TSWV genomes in the GenBank (accession numbers: NC_002050 to NC_002052 and KT160280 to KT160282, accessed on 05 May 2021). Primer pairs, annealing temperatures, and amplicon sizes are listed in Table 5. The reaction mix consisted of 25 μL of GoTaq Green Master Mix (Promega Corporation, Madison, WI, USA), 2.5 μL (0.5 μM) of each forward and reverse primer, 5 μL of synthesized cDNA, and 15 μL nuclease-free water. The PCR program started with an initial activation step at 95 °C for 2 min followed by 35 cycles of amplification and a final extension step at 72 °C for 5 min. The amplification cycle included denaturation at 94 °C for 1 min, annealing at the primer-specific temperature for 45 s, and extension at 72 °C for 50 s (N, NSs, NSm, Gn/Gc genes) or 90 s (RdRp gene). The presence of targeted amplicons in the PCR product was visualized by agarose gel (1%) electrophoresis. The PCR product was purified using the GeneJET PCR purification kit (Thermo Scientific™, Thermo Fisher Scientific, Waltham, MA, USA) according to the manufacturer’s protocol. Purified PCR products were sequenced in both directions using the SimpleSeq™ DNA sequencing service from Eurofins Genomics (Eurofins MWG Operon Inc., Louisville, KY, USA). Consensus sequences were assembled from sequences of both directions and edited in Geneious Prime^®^ (version 2019.2.3) [93]. Sequences of the full-length N gene and partial NSs, NSm, Gn/Gc, and RdRp genes obtained in this study were deposited in the GenBank with accession numbers MW519186–MW519472 (Appendix A Table A1).

### 4.3. TSWV Isolates and Sequence Alignments

Nucleotide sequences were aligned using Clustal W with default settings in Geneious Prime^®^. Sequence alignments were manually corrected when necessary. For the N gene, a nucleotide alignment of 150 TSWV isolates collected in Georgia, including 59 isolates collected in this study, 82 isolates collected in 2010 (GenBank accession numbers: HQ40603-HQ406984, accessed on 05 may 2021), and nine isolates collected in 1998 (GenBank accession numbers: AF048714-AF018716 and AF064469-AF064474, accessed on 05 may 2021), were used for the analysis. The nine isolates were collected from peanut and solanaceous crops in Tift County in 1998 [46]; while the 80 isolates were collected from peanut in ten counties in Georgia, and two isolates were from solanaceous crops collected in Tift County in 2010 [40]. Sequence alignments of partial NSs and GnGc genes were obtained from 59 TSWV isolates and used for the analysis. Sequence alignments for the partial NSm gene and RdRp genes were obtained from 58 and 52 TSWV isolates, respectively.

### 4.4. Phylogenetic Analysis and Tree Construction

Phylogenetic analysis was conducted for each TSWV gene using DNA sequence alignments. The best-fitting nucleotide substitution model for each dataset (sequence alignment for each gene) was selected using ModelTest-NG [94] based on Bayesian Information Criterion (BIC) before phylogenetic analysis. Bayesian analysis was performed in MrBayes v3.2.7 [95], and phylogenetic trees were constructed using data output from Bayesian MCMC analysis using Interactive Tree of Life [96].

### 4.5. Genetic Diversity, Test of Neutrality, and Identification of Selection

Genetic variation in each gene among TSWV isolates was evaluated. The nucleotide diversity (π, the pairwise average number of nucleotide differences per site) [97] and the Watterson’s estimator (θ_w_, a measure of the population mutation rate based on segregating sites) [97,98] were estimated using DnaSP (v6.12.03) [99]. Population neutrality, the hypothesis of all mutation being selectively neutral, was tested by Tajima’s D [98] and Fu and Li’s D * and F * statistics [99]. Tajima’s D test statistic is based on the relationship between the number of segregating sites (total number of mutations) and the pairwise average number of nucleotide differences; the significance of Tajima’s D statistic is determined by a two-tailed test under the beta distribution [100]. The Fu and Li’s D * and F * test statistics are based on the relationship between numbers of external and internal mutations in the genealogy of sequences from a population. D * statistic measures the differences between the number of singletons (sites of nucleotide variants that only appear in one sequence of the population) and total number of mutations. F * statistic assesses the differences between the singleton number and the pairwise average number of nucleotide differences [99]. The statistical significances of Fu and Li’s D * and F * statistics were determined using critical values obtained from simulated distribution of each statistic over the interval of mutation rate (θ) from 2 to 20 [101]. When Tajima’s D and Fu and Li’s D * and F * statistics were indistinguishable from zero, neutral variation was implied, indicating that there was no evidence for changes in population size or directional selections. Negative statistics indicated the occurrence of population expansion or purifying selection and an excess of rare mutations, while positive statistics implied a population bottleneck with deficiency of rare mutations.

Selection pressure on codons of TSWV genes were assessed using the Fast, Unconstrained Bayesian AppRoximation (FUBAR) test in Hypothesis Testing using Phylogenies (HyPhy) software package. FUBAR uses a Bayesian approach to infer non-synonymous (dN) and synonymous (dS) substitution rate on a per codon basis [102]. Positive selection was inferred when the posterior non-synonymous substitution rate (β) was higher than synonymous substitution rate (α) at a given codon site; in contrast, negative (purifying) selection was indicated when the substitution rate was higher for synonymous substitution than non-synonymous substitution. Significance of selection was determined by the posterior probability of the difference between the rates of synonymous and non-synonymous substitution with a significant level of Prob = 0.900.

### 4.6. Test of Gene Flow and Population Differentiation

Nucleotide-based statistics, including Ks, Kst, and Snn [103], were computed using the Gene Flow and Genetic Differentiation analysis module in DnaSP v.6. Significance tests for nucleotide-based statistics were fulfilled by randomization (permutation) tests with 1000 replications. The extent of genetic differentiation between TSWV populations was examined by evaluating the fixation index (Fst) [104].

### 4.7. Thrips Feeding Injury and Survival

**Figure 6:**
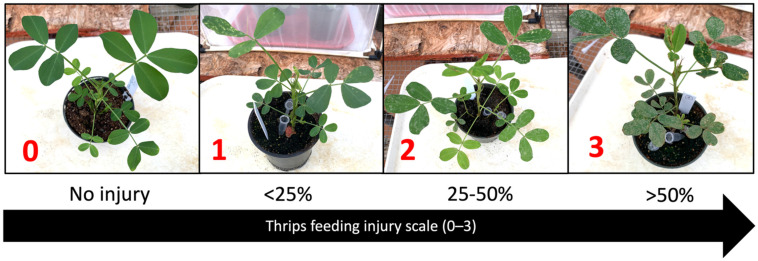
Thrips feeding injury scale of 0–3, where 0 represents no injury, and 1 to 3 represent <25%, 25–50%, and >50% leaf area of individual leaflets having feeding scars, respectively.

The effect of TSWV-resistant peanut cultivars on thrips feeding injury and survival was evaluated through replicated no-choice tests in a greenhouse. *Frankliniella fusca* from a laboratory colony established in 2009 at the University of Georgia were used for the experiment; *F. fusca* were reared on greenhouse-grown Georgia Green leaflets maintained in small Petri dishes (35 mm diameter) with wet cotton rounds. The colony was maintained in a growth chamber (Thermo Fisher Scientific, Waltham, MA, USA) at 25–30 °C with an L14:D10 photoperiod [16,105,106,107]. Six peanut cultivars with varying levels of TSWV susceptibility were used: Florunner, Georgia Green, Georgia-06G, Georgia-12Y, Georgia-16HO, and Tifguard. For each cultivar, six peanut seedlings (at two-node stage) were placed in a thrips-proof cage (Megaview Science Co., Taichung, Taiwan). Ten thrips (adult female thrips up to three days old) were released on each peanut plant. A trace of pine pollen was dusted on the surface of leaves as a supplement [108]. Plants were evaluated for thrips injury, and surviving thrips were counted at three-day intervals for up to 30 days after thrips release. Thrips larvae and adults on leaves and stems of the plants were counted. Thrips feeding injury was rated on a scale of 0–3, where 0 represented no injury, and 1 to 3 represented <25%, 25–50%, and >50% leaf area of individual leaflets having feeding scars, respectively (Figure 6). Feeding damage index (FDI) was calculated based on a formula originally proposed by Maris et al. (2003) [44] and modified by Sundaraj et al. (2014) [40]: FDI = (number of leaflets with feeding injury/total number of leaflets in a plant) x injury rating. The experiment was conducted two times (*n* = 12 per cultivar).

Thrips count and feeding damage index data were pooled from experiments for statistical analysis. Thrips count data were subjected to generalized linear mixed model analysis using the PROC GLIMMIX procedure with the negative binomial distribution and the log link function in SAS (SAS Enterprise 9.4, SAS Institute, Cary, NC, USA). Peanut cultivar served as a fixed effect, and experiment and replication were random effects. Least square means (LS-means) were used for multiple comparisons at significant level of α = 0.05 with Tukey–Kramer adjustment to determine significant differences between peanut cultivars. Feeding damage index data were subjected to the Wilcoxon Score tests using the PROC NPAR1WAY procedure in SAS, and the significance of cultivar effect was determined by Kruskal–Wallis test (one-way ANOVA test for the Wilcoxon Score tests) at α = 0.05. When the cultivar effect was significant on feeding damage index, multiple pairwise Wilcoxon two-sample tests and Kruskal–Wallis tests were conducted among cultivars.

### 4.8. Thrips Development, Reproduction, and Oviposition

The effect of TSWV-resistant peanut cultivars on thrips developmental time, number of adult thrips produced, and number of eggs laid were evaluated through microcosm experiments. Thrips and peanut cultivars used in these experiments were the same as those used for evaluating thrips injury and survival. Six Munger cages [109] were set up for each cultivar, and the experiment was conducted two times (*n* = 12 per cultivar). Ten thrips (adult female thrips up to three days old) were transferred onto two peanut leaflets with a trace of pollen in a Munger cage using a fine paintbrush. Adult female thrips were removed from the Munger cages after 72 h. The cages were monitored daily under a dissecting microscope (40×) (MEIJI TECHNO, Santa Clara, CA, USA). Adult thrips emerging from each cage were counted at 24 h intervals and removed from the cage. Developmental time (adult to adult) for each thrips emerged was recorded. The adult thrips removed from the Munger cages were further used for oviposition on peanut leaflets of the same cultivar on which they developed. Five female adult thrips (up to three days old) were transferred onto two leaflets with a trace of pollen in a small Petri dish with a wet cotton round. Cages were secured by rubber bands to avoid thrips escape, and adult thrips were allowed to oviposit for 72 h and removed. During the experiments, all cages were maintained in a growth chamber (Thermo Fisher Scientific, Waltham, MA) at 25–30 °C with an L14:D10 photoperiod. Subsequently, peanut leaflets were stained for egg counting using a staining method described by Ben-Mahmoud et al. (2018) [110]. Peanut leaflets were immersed in McBride’s solution (0.2% acid fuchsin in 1:1 ethanol:glacial acetic acid) and shaken on a benchtop orbital shaker (MAXQ4450, Thermo Scientific, Waltham, MA, USA) at a low speed (145 rpm) for 24 h. Leaflets were then transferred to clean vials and soaked in a de-staining solution (1:1:1 lactic acid:glycerol:water). After being shaken for 3 h, the vials were moved to an incubator (Isotemp Oven Model 630G, Fisher Scientific, Waltham, MA, USA) at 80 °C and incubated for 24 h. Leaflets were allowed to cool at room temperature, and thrips eggs (partially or fully embedded in leaf tissues) were stained red and counted under a dissecting microscope (100×) (MEIJI TECHNO, Santa Clara, CA, USA).

Adult thrips counts, thrips developmental time from adult to adult, and egg counts were pooled across experiments for statistical analysis. Adult thrips counts and egg counts were subjected to generalized linear mixed model analysis using the PROC GLIMMIX procedure with the Poisson distribution and the log link function in SAS. Peanut cultivars served as a fixed effect, while experiment and replication were random effects. LS-means were used for multiple comparisons to determine significant differences between peanut cultivars. For adult thrips counts, Tukey–Kramer adjustment was applied, while Fisher’s LSD was used for egg count data. Median developmental time of thrips was subjected to Wilcoxon Score tests using the PROC NPAR1WAY procedure in SAS, and the significance of the cultivar effect was determined by Kruskal–Wallis test at α = 0.05.

## Figures and Tables

**Table 1:** Summary of parameters for genetic variation and neutrality test statistics for five TSWV genes.

Gene	Subgroups	N ^o^	S ^p^	π (SD) ^q^	θ_w_^r^	Tajima’s D ^s^	Fu and Li’s D *^, t^	Fu and Li’s F *^, u^
N	Overall	150	162	0.0083 (0.0005)	0.0373	−2.550 ***^, v^	−5.927 **	−5.232 **
	GA-1998 ^w^	9	27	0.0102 (0.0021)	0.0128	−1.000	−1.116	−1.220
	GA-2010 ^x^	82	100	0.0075 (0.0006)	0.0259	−2.41 **	−5.315 **	−4.950 **
	2010-S ^x^	10	28	0.0098 (0.0017)	0.0127	−1.111	−1.012	−1.170
	2010-R ^x^	72	89	0.0072 (0.0006)	0.0236	−2.390 **	−4.843 **	−4.634 **
	GA-New ^y^	59	93	0.0076 (0.0006)	0.0258	−2.484 **	−4.350 **	−4.348 **
	New-S ^y^	10	29	0.0086 (0.0016)	0.0132	−1.6560	−1.903.	−2.078.
	New-R ^y^	49	79	0.0075 (0.0006)	0.0228	−2.428 **	−4.113 **	−4.168 **
	After 2010 ^z^	141	152	0.0076 (0.0004)	0.0354	−2.574 ***	−5.648 **	−5.089 **
	After 2010-S ^z^	20	48	0.0094 (0.0013)	0.0174	−1.8545 *	−2.263	−2.495
	After 2010-R ^z^	121	137	0.0074 (0.0004)	0.0328	−2.5791 ***	−5.334 **	−4.933 **
NSs	Overall	59	108	0.0072 (0.0005)	0.0268	−2.586 ***	−4.505 **	−4.507 **
	Susceptible	10	29	0.0072 (0.0011)	0.0118	−1.853 *	−2.183 *	−2.370 *
	Resistant	49	97	0.0072 (0.0005)	0.0250	−2.573 ***	−4.517 **	−4.534 **
NSm	Overall	58	91	0.0126 (0.0006)	0.0292	−2.047 *	−3.335 *	−3.401 **
	Susceptible	10	30	0.0126 (0.0011)	0.0158	−0.969	−1.113	−1.215
	Resistant	48	82	0.0126 (0.0007)	0.0275	−1.979 *	−3.013 *	−3.142 *
Gn/Gc	Overall	59	147	0.0116 (0.0008)	0.0419	−2.620 ***	−5.042 **	−4.910 **
	Susceptible	10	34	0.0097 (0.0013)	0.0159	−1.890 *	−2.241 **	−2.431 **
	Resistant	49	132	0.0119 (0.0009)	0.0392	−2.571 ***	−4.910 **	−4.824 **
RdRp	Overall	52	226	0.0123 (0.0005)	0.0353	−2.395 **	−4.199 **	−4.202 **
	Susceptible	9	65	0.0132 (0.0014)	0.0169	−1.181	−1.304	−1.432
	Resistant	43	202	0.0121 (0.0006)	0.0330	−2.378 **	−3.715 **	−3.851 **

^o^ Number of sequences in the subgroup; ^p^ number of segregating sites; ^q^ nucleotide diversity with standard deviation in parentheses; estimates can range from 0 to 0.100; ^r^ Watterson’s estimator per site based on number of segregating sites; ^s^ Tajima’s D compares the nucleotide diversity with the proportion of segregating sites; a negative value provides evidence for population expansion and/or purifying selection at the locus; ^t^ Fu and Li’s D * is based on the differences between the number of singletons and the total number of mutations; a negative value provides evidence for population expansion and/or purifying selection at the locus; ^u^ Fu and Li’s F * is based on the differences between the number of singletons and the average number of nucleotide differences between sequences; a negative value provides evidence for population expansion and/or purifying selection at the locus; ^v^ significance of the value is denoted as “*”, *p <* 0.1; “*”, *p <* 0.05, “**”, *p <* 0.01; “***”, *p <* 0.001; ^w^ N gene sequences of TSWV isolates from [46]; ^x^ N gene sequences of TSWV isolates from [40]; ^y^ N gene sequences of TSWV isolates collected in this study from 2016 to 2019 and subgroups of isolates from susceptible (S) and resistant (R) cultivars; ^z^ N gene sequences of TSWV isolates collected after 2010 from Sundaraj et al. [40] and this study with subgroups of isolates from susceptible (S) and resistant (R) cultivars. Whole gene sequences were used for the N gene, and partial sequences were used for all others.

**Table 2:** Summary of codon sites with amino acid substitutions in five TSWV genes.

Gene	Codon Site ^u^	α ^v^	Β ^w^	β-α ^x^	Prob [α < β] ^y^	Amino Acid Changes ^z^
N	7	1.508	6.379	4.871	0.902 *	T→I, F
	8	0.799	0.833	0.034	0.545	K→M
	10	0.531	1.180	0.648	0.738	S→N
	18	0.542	0.681	0.139	0.583	G→S
	19	0.771	0.806	0.036	0.545	K→I
	40	2.687	8.793	6.105	0.828	G→D, E
	174	0.544	0.877	0.333	0.625	Y→C
	222	0.994	2.559	1.565	0.726	S→C
NSs	16	1.128	3.666	2.538	0.667	Q→K
	53	1.049	1.253	0.204	0.582	S→T
	98	1.066	1.244	0.178	0.580	I→V
	114	1.035	1.158	0.122	0.576	T→A
	152	1.144	1.174	0.030	0.567	E→K
	193	0.849	1.514	0.666	0.623	N→D
	243	5.451	11.60	6.149	0.709	T→I, A
	257	1.051	1.099	0.048	0.569	P→T
	281	0.732	2.528	1.796	0.684	L→M
NSm	124	1.127	1.462	0.335	0.592	K→R
	210	1.056	5.211	4.155	0.824	K→I, T
Gn/Gc	51	0.656	0.971	0.315	0.609	N→S
	82	1.018	1.138	0.120	0.561	Y→S
	135	1.401	4.190	2.789	0.753	S→N
	139	0.656	0.971	0.315	0.609	N→S
	165	0.883	1.234	0.352	0.588	L→V
	223	0.619	2.307	1.688	0.790	D→N
RdRp	51	0.792	3.945	3.153	0.863	V→I
	**149**	1.006	6.957	5.951	0.905 *	K→R
	**207**	0.902	11.777	10.875	0.959 *	Q→R
	208	0.967	1.051	0.085	0.545	N→D
	211	0.689	0.905	0.217	0.580	I→V
	244	0.796	0.900	0.104	0.555	E→K
	280	0.699	1.067	0.368	0.596	N→D
	331	2.785	5.005	2.220	0.639	I→A
	341	0.699	1.061	0.362	0.595	N→S
	**368**	0.865	16.328	15.463	0.992 *	G→R, E, A
	**397**	0.665	9.774	9.109	0.912 *	Q→L, H
	414	0.708	0.791	0.082	0.557	S→T

^u^ Codon positions with overabundance of non-synonymous substitutions; ^v^ mean posterior synonymous substitution rate at a codon site; ^w^ mean posterior non-synonymous substitution rate at a codon site; ^x^ mean posterior β-α; a positive value indicates an overabundance of non-synonymous substitutions; ^y^ posterior probability of positive selection at a codon site; significance was determined at Prob [α < β] > 0.900; ^z^ codon changes due to substitutions. Whole gene sequences were used for the N gene, and partial sequences were used for all others.

**Table 3:** Summary of parameter estimates and test statistics for population differentiation.

Gene	Comparison ^r^	Ks ^s^	Kst ^s^	*p*(Ks/Kst) ^r^	Snn ^t^	*p* (Snn) ^r^	Fst ^u^
N	GA-1998 ^v^ vs. GA-2010 ^w^	6.0059	0.0782	**<0.001**	0.9923	**<0.001**	0.2915
	GA-1998 ^v^ vs. GA-2016 to 2019 ^x^	6.1783	0.1230	**<0.001**	1.0000	**<0.001**	0.3493
	GA-1998 ^v^ vs. After 2010 ^y^	6.0517	0.0559	**<0.001**	0.9953	**<0.001**	0.3126
	GA-2010 ^w^ vs. GA-2016 to 2019 ^x^	5.8415	0.0151	**<0.001**	0.6490	**<0.001**	0.0302
	GA-2010 S vs. R ^z^	5.8045	−0.0020	0.637	0.8438	**0.041**	−0.0081
	GA-2016 to 2019 S vs. R ^z^	5.9383	−0.0050	0.923	0.6825	0.776	−0.0168
	After 2010 S vs. R ^z^	5.9385	−0.0013	0.722	0.7590	0.483	−0.0048
NSs	S vs. R ^z^	6.2446	−0.0052	0.982	0.6059	0.886	−0.0184
NSm	S vs. R ^z^	8.4877	−0.0022	0.559	0.6997	0.614	−0.0077
GnGc	S vs. R ^z^	8.7208	0.0012	0.302	0.6439	0.779	0.0044
RdRp	S vs. R ^z^	17.3748	0.0024	0.244	0.7212	0.606	0.0080

^r^ Comparison between population subgroups of TSWV isolates; ^s^ Ks and Kst are nucleotide-based test statistics of population differentiation; ^t^ test statistic Snn is independent of sample size and diversity; a value close to 1 indicates differentiation; ^u^ Fst, a nucleotide-based test statistic, determines the extent of genetic differentiation; ^v^ N gene sequences of TSWV isolates from [46]; ^w^ N gene sequences of TSWV isolates from Sundaraj et al. [40]; ^x^ N gene sequences of TSWV isolates collected in this study from 2016 to 2019; ^y^ N gene sequences of TSWV isolates collected after 2010 from Sundaraj et al. [40] and this study; ^z^ subgroups of isolates from susceptible (S) and resistant (R) cultivars. Whole gene sequences were used for the N gene, and partial sequences were used for all others.

**Table 4:** Information regarding peanut samples from which TSWV isolates were collected and sequenced.

Type	Cultivar	Year	Location	Reference	Susceptibility Subgroups ^z^
Runner	AUNPL-17	2017, 2018, 2019	Tifton	N/A	Resistant
Runner	FloRun 107	2016, 2017	Tifton	[74]	Resistant
Runner	FloRun 331	2017, 2018, 2019	Tifton	[75]	Resistant
Runner	Florunner	2018, 2019	Tifton	[76]	Susceptible
Runner	Georgia Green	2016, 2017, 2018, 2019	Tifton, Attapulgus	[77]	Susceptible
Runner	Georgia-06G	2016, 2017, 2018, 2019	Tifton, Attapulgus	[78]	Resistant
Runner	Georgia-12Y	2018, 2019	Tifton	[79]	Resistant
Runner	Georgia-13M	2016, 2017, 2018	Tifton	[80]	Resistant
Runner	Georgia-14N	2016, 2017, 2018, 2019	Tifton	[81]	Resistant
Runner	Georgia-16HO	2017, 2018, 2019	Tifton, Attapulgus	[82]	Resistant
Runner	Georgia-18RU	2019	Tifton	[83]	Resistant
Runner	Tifguard	2016, 2017, 2018, 2019	Tifton	[84]	Resistant
Runner	TifNV-High O/L	2017, 2018, 2019	Tifton	[85]	Resistant
Runner	TUFRunner 157	2017	Tifton	N/A	Resistant
Runner	TUFRunner 297	2017, 2018, 2019	Tifton	[86]	Resistant
Runner	TUFRunner 511	2017, 2018	Tifton, Attapulgus	[87]	Resistant
Runner	TUFRunner 727	2016, 2017	Tifton	N/A	Resistant
Spanish	Georgia-04S	2016	Tifton	[88]	Resistant
Valencia	New Mexico Valencia C	2019	Tifton	[89]	Susceptible
Virginia	Bailey	2019	Tifton	[90]	Resistant
Virginia	Georgia-11J	2017, 2019	Tifton, Attapulgus	[91]	Resistant
Virginia	Gregory	2019	Tifton	[92]	Susceptible

^z^ Grouping for TSWV-susceptible and -resistant subgroups for analyses of population genetics based on relative TSWV susceptibility. N/A- indicates not available.

**Table 5:** Primer pairs used for PCR.

RNA Fragments	Gene	Primer Pairs	Direction	Sequence (5′ -> 3′)	Annealing Temperature (°C)	Amplicon Size (bp) ^y^
L	RdRp	L6885	F	CTGTCCTCATTGTCGTGCCT	58	1416
		L8403	R	CAACTAACGCCACCCCTGAT		
M	NSm	M290	F	ACATCTTCCTTTGGAACCTA	53	672
		M962	R	CCTCTTCTTCTCCAACTGAT		
	GnGc	M2565	F	ACCAAGCTTCTTCACATCC	58	756
		M3320	R	TTTATGTTCCAGGCTGTCC		
S	NSs	S574	F	GTCTTGTGTCAAAGAGCATACCTATAA	58	869
		S1433	R	TGATCCCGCTTAAATCAAGCT		
	N ^z^	S2057	F	TTAAGCAAGTTCTGTGAG	52	777
		S2833	R	ATGTCTAAGGTTAAGCTC		

^y^ Expected amplicon size for each primer pair from PCR based on reference sequences (NC_002050 to NC_002052 and KT160280 to KT160282); ^z^ primers obtained from [46].

## Data Availability

The data generated as part of this study were submitted to the NCBI GenBank database (https://www.ncbi.nlm.nih.gov/genbank/ accessed on 22 October 2021). The deposited sequences can be accessed with accession numbers MW519186–MW519472.

## Supplementary Materials

The following are available online at https://www.mdpi.com/article/10.3390/pathogens10111418/s1, Table S1: Mean and standard error of thrips number survived on peanut plants.

## Appendix A

**Table A1:** Gene sequences of TSWV isolates collected from susceptible and resistant peanut cultivars with GenBank accession numbers.

N		NSs		NSm		Gn/Gc		RdRp	
Isolate ID	GenBank Accession	Isolate ID	GenBank Accession	Isolate ID	GenBank Accession	Isolate ID	GenBank Accession	Isolate ID	GenBank Accession
FloRun331 2018	MW519186	Tifguard 2017	MW519245	GeorgiaGreen 2018 A	MW519304	Georgia14N 2016	MW519362	Georgia04S 2016	MW519421
GeorgiaGreen 2019 A	MW519187	Florunner 2018 J	MW519246	FloRun331 2018 L	MW519305	Georgia12Y 2019	MW519363	Tifguard 2018 J	MW519422
Georgia12Y 2018 L	MW519188	Georgia04S 2016	MW519247	GeorgiaGreen 2016	MW519306	NMValenciaC 2019	MW519364	Florunner 2018 J	MW519423
AUNPL17 2017	MW519189	Georgia14N 2017	MW519248	Georgia13M 2018 J	MW519307	FloRun107 2016	MW519365	Florunner 2019	MW519424
Georgia13M 2016	MW519190	Georgia18RU 2019	MW519249	GeorgiaGreen 2018 J	MW519308	TUFRunner157 2017	MW519366	Georgia06G 2016	MW519425
Georgia16HO 2019	MW519191	GeorgiaGreen 2019 A	MW519250	Georgia06G 2018 A	MW519309	Bailey 2019	MW519367	GeorgiaGreen 2016	MW519426
Florunner 2018 J	MW519192	Georgia12Y 2019	MW519251	TUFRunner511 2018 L	MW519310	Gregory 2019	MW519368	TUFRunner297 2018 L	MW519427
Georgia16HO 2017	MW519193	Georgia16HO 2017	MW519252	NMValenciaC 2019	MW519311	Georgia13M 2017	MW519369	TUFRunner727 2017	MW519428
Tifguard 2017	MW519194	Georgia16HO 2019	MW519253	Georgia06G 2018 J	MW519312	Georgia06G 2019 A	MW519370	GeorgiaGreen 2018 A	MW519429
FloRun107 2017	MW519195	Georgia06G 2019	MW519254	Bailey 2019	MW519313	GeorgiaGreen 2016	MW519371	Georgia06G 2019 A	MW519430
Georgia06G 2019	MW519196	NMValenciaC 2019	MW519255	Gregory 2019	MW519314	GeorgiaGreen 2018 A	MW519372	Georgia11J 2017	MW519431
Georgia14N 2018 J	MW519197	Georgia14N 2018 J	MW519256	Georgia06G 2019 A	MW519315	TifNVHOL 2017	MW519373	Georgia16HO 2018 P	MW519432
TUFRunner297 2017	MW519198	TUFRunner297 2017	MW519257	Georgia14N 2016	MW519316	Georgia16HO 2018 A	MW519374	Tifguard 2016	MW519433
FloRun107 2016	MW519199	Georgia12Y 2018 L	MW519258	AUNPL17 2017	MW519317	Georgia06G 2018 J	MW519375	TUFRunner511 2018 P	MW519434
Georgia14N 2017	MW519200	TUFRunner511 2018 L	MW519259	Georgia04S 2016	MW519318	TUFRunner511 2018 L	MW519376	FloRun107 2017	MW519435
Georgia18RU 2019	MW519201	Georgia06G 2019 A	MW519260	AUNPL17 2018 J	MW519319	Georgia11J 2019 A	MW519377	GeorgiaGreen 2019	MW519436
Georgia04S 2016	MW519202	Georgia13M 2016	MW519261	TifNVHOL 2019	MW519320	Georgia13M 2018 J	MW519378	TUFRunner297 2019	MW519437
Georgia06G 2017	MW519203	TUFRunner511 2017	MW519262	TUFRunner511 2018 A	MW519321	GeorgiaGreen 2018 J	MW519379	Georgia14N 2016	MW519438
Georgia06G 2016	MW519204	FloRun107 2016	MW519263	GeorgiaGreen 2019	MW519322	Georgia06G 2017	MW519380	Georgia16HO 2018 A	MW519439
AUNPL17 2018 J	MW519205	TUFRunner511 2018 A	MW519264	GeorgiaGreen 2017	MW519323	Georgia14N 2019	MW519381	Georgia14N 2017	MW519440
TUFRunner331 2019	MW519206	AUNPL17 2017	MW519265	Georgia13M 2016	MW519324	TUFRunner727 2017	MW519382	TifNVHOL 2018 J	MW519441
Gregory 2019	MW519207	GeorgiaGreen 2016	MW519266	TUFRunner297 2019	MW519325	FloRun331 2018 L	MW519383	FloRun331 2018 L	MW519442
Georgia11J 2019 A	MW519208	Georgia16HO 2018 P	MW519267	Georgia16HO 2018 P	MW519326	Georgia06G 2018 A	MW519384	Georgia12Y 2018 L	MW519443
Bailey 2019	MW519209	FloRun331 2018 L	MW519268	TifNVHOL 2018 J	MW519327	Georgia04S 2016	MW519385	TUFRunner511 2018 L	MW519444
Georgia11J 2017	MW519210	AUNPL17 2019	MW519269	FloRun107 2017	MW519328	Georgia18RU 2019	MW519386	Georgia06G 2019	MW519445
TifNVHOL 2019	MW519211	Tifguard 2019	MW519270	TUFRunner297 2017	MW519329	Georgia06G 2019	MW519387	AUNPL17 2017	MW519446
Tifguard 2016	MW519212	Georgia06G 2018 A	MW519271	Georgia13M 2017	MW519330	TUFRunner511 2018 A	MW519388	TUFRunner297 2017	MW519447
Georgia13M 2018 J	MW519213	Georgia06G 2018 J	MW519272	TUFRunner511 2017	MW519331	Georgia06G 2016	MW519389	FloRun331 2017	MW519448
GeorgiaGreen 2018 J	MW519214	Georgia14N 2019	MW519273	FloRun331 2019	MW519332	TifNVHOL 2019	MW519390	FloRun107 2016	MW519449
Tifguard 2018 J	MW519215	Georgia11J 2019 A	MW519274	AUNPL17 2019	MW519333	AUNPL17 2018 J	MW519391	Georgia12Y 2019	MW519450
TUFRunner727 2016	MW519216	Georgia11J 2017	MW519275	Tifguard 2019	MW519334	Florunner 2019	MW519392	Georgia18RU 2019	MW519451
Georgia06G 2018 A	MW519217	Tifguard 2016	MW519276	Florunner 2018 J	MW519335	Georgia14N 2018 J	MW519393	TifNVHOL 2017	MW519452
TUFRunner297 2018 L	MW519218	Florunner 2019	MW519277	TUFRunner511 2018 P	MW519336	Georgia16HO 2018 P	MW519394	TUFRunner727 2016	MW519453
AUNPL17 2019	MW519219	GeorgiaGreen 2017	MW519278	Georgia14N 2018 J	MW519337	TUFRunner297 2017	MW519395	Georgia16HO 2017	MW519454
Tifguard 2019	MW519220	TUFRunner297 2019	MW519279	TUFRunner297 2018 L	MW519338	FloRun107 2017	MW519396	TUFRunner157 2017	MW519455
TUFRunner511 2018 P	MW519221	TifNVHOL 2018 J	MW519280	GeorgiaGreen 2019 A	MW519339	TUFRunner511 2018 P	MW519397	Georgia06G 2018 J	MW519456
Florunner 2019	MW519222	GeorgiaGreen 2019	MW519281	Georgia16HO 2019	MW519340	AUNPL17 2019	MW519398	Georgia16HO 2019	MW519457
Georgia14N 2016	MW519223	Georgia13M 2017	MW519282	Tifguard 2016	MW519341	Tifguard 2019	MW519399	NMValenciaC 2019	MW519458
Georgia14N 2019	MW519224	TUFRunner157 2017	MW519283	Florunner 2019	MW519342	Georgia13M 2016	MW519400	FloRun331 2019	MW519459
GeorgiaGreen 2017	MW519225	Bailey 2019	MW519284	Georgia18RU 2019	MW519343	TifNVHOL 2018 J	MW519401	TUFRunner511 2017	MW519460
Georgia16HO 2018 A	MW519226	Gregory 2019	MW519285	Georgia06G 2016	MW519344	TUFRunner297 2019	MW519402	Georgia13M 2018 J	MW519461
GeorgiaGreen 2018 A	MW519227	TifNVHOL 2019	MW519286	TUFRunner727 2016	MW519345	GeorgiaGreen 2019	MW519403	GeorgiaGreen 2018 J	MW519462
TifNVHOL 2017	MW519228	Georgia16HO 2018 A	MW519287	Tifguard 2017	MW519346	GeorgiaGreen 2017	MW519404	AUNPL17 2019	MW519463
Georgia06G 2018 J	MW519229	Tifguard 2018 J	MW519288	Georgia16HO 2017	MW519347	AUNPL17 2017	MW519405	Tifguard 2019	MW519464
Georgia13M 2017	MW519230	TUFRunner297 2018 L	MW519289	FloRun331 2017	MW519348	TUFRunner727 2016	MW519406	Georgia14N 2018 J	MW519465
TUFRunner157 2017	MW519231	TifNVHOL 2017	MW519290	Georgia12Y 2018 L	MW519349	Tifguard 2017	MW519407	Georgia14N 2019	MW519466
TifNVHOL 2018 J	MW519232	Georgia06G 2016	MW519291	Tifguard 2018 J	MW519350	Georgia16HO 2017	MW519408	GeorgiaGreen 2019 A	MW519467
GeorgiaGreen 2019	MW519233	FloRun331 2017	MW519292	Georgia06G 2019	MW519351	FloRun331 2017	MW519409	TifNVHOL 2019	MW519468
TUFRunner297 2019	MW519234	TUFRunner727 2016	MW519293	Georgia11J 2017	MW519352	Georgia11J 2017	MW519410	GeorgiaGreen 2017	MW519469
GeorgiaGreen 2016	MW519235	Georgia14N 2016	MW519294	Georgia14N 2017	MW519353	Georgia14N 2017	MW519411	Tifguard 2017	MW519470
TUFRunner331 2017	MW519236	Georgia06G 2017	MW519295	FloRun107 2016	MW519354	Georgia12Y 2018 L	MW519412	Georgia06G 2017	MW519471
TUFRunner727 2017	MW519237	FloRun331 2019	MW519296	Georgia06G 2017	MW519355	Tifguard 2018 J	MW519413	Georgia13M 2017	MW519472
Georgia06G 2019 A	MW519238	TUFRunner511 2018 P	MW519297	TifNVHOL 2017	MW519356	Georgia16HO 2019	MW519414		
Georgia16HO 2018 P	MW519239	AUNPL17 2018 J	MW519298	Georgia11J 2019 A	MW519357	Tifguard 2016	MW519415		
NMValenciaC 2019	MW519240	Georgia13M 2018 J	MW519299	TUFRunner727 2017	MW519358	TUFRunner297 2018 L	MW519416		
TUFRunner511 2018 L	MW519241	GeorgiaGreen 2018 J	MW519300	Georgia16HO 2018 A	MW519359	GeorgiaGreen 2019 A	MW519417		
Georgia12Y 2019	MW519242	GeorgiaGreen 2018 A	MW519301	Georgia14N 2019	MW519360	FloRun331 2019	MW519418		
TUFRunner511 2018 A	MW519243	TUFRunner727 2017	MW519302	TUFRunner157 2017	MW519361	Florunner 2018 J	MW519419		
TUFRunner511 2017	MW519244	FloRun107 2017	MW519303			TUFRunner511 2017	MW519420		
