# Supplementary material for: Impact of Host Resistance to Tomato Spotted Wilt Orthotospovirus in Peanut Cultivars on Virus Population Genetics and Thrips Fitness
Source: Pathogens. 2021 Nov 1;10(11):1418. doi: 10.3390/pathogens10111418 (PMC8625697; doi:10.3390/pathogens10111418)
Supplement: Supplementary file 1 [file pathogens-10-01418-s001.zip › pathogens-1425289-supplementary.pdf]

Supplementary Materials:

Table S1. Mean and standard error of thrips that survived on peanut plants.

**Cultivar**[x]	**Thrips number (mean ± SE)**[y]									
	3d	6d	**9d**	**12d**	15d	18d	21d	24d	27d	30d
Florunner	2.42 ± 0.47	2.58 ± 0.54	20.08 ±7.38 AB	45.50 ± 6.44 B	47.5 ± 5.21	33.92 ± 4.27	34.08 ± 3.87	29.17 ± 2.53	38.92 ± 3.49	66.58 ± 7.49
Georgia Green	2.17 ± 0.24	3.17 ± 0.39	16.75 ± 3.66 AB	83.83 ± 11.02 A	72.75 ± 7.22	46.17 ± 5.35	39.67 ± 4.48	40.5 ± 7.24	49.00 ± 5.19	60.08 ± 7.07
Georgia-06G	3.08 ± 0.26	2.67 ± 0.36	13.42 ± 3.36 B	59.75 ± 4.49 AB	74.45 ± 8.84	53.36 ± 8.43	47.36 ± 7.01	38.27 ± 5.27	42.45 ± 6.4	68.36 ± 9.99
Georgia-12Y	3.08 ± 0.29	4.00 ± 0.41	3.17 ± 0.34 C	41.25 ± 5.50 B	68.42 ± 5.73	55.67 ± 5.22	46.50 ± 2.99	41.92 ± 4.63	49.33 ± 6.25	56.33 ± 7.86
Georgia-16HO	2.17 ± 0.49	2.25 ± 0.51	24.58 ± 4.05 AB	64.50 ± 6.31 AB	55.25 ± 6.27	42.42 ± 4.65	40.00 ± 6.02	29.83 ± 5.38	41.5 ± 5.57	74.50 ± 9.38
Tifguard	2.50 ± 0.44	3.08 ± 0.56	29.75 ± 4.19 A	62.42 ± 7.36 AB	62.92 ± 9.5	48.17 ± 7.78	37.17 ± 5.59	28.00 ± 3.34	43.42 ± 7.3	64.58 ± 13.33
Type III tests[z]										
F value (df)	0.53 (5, 583)	0.92 (5, 583)	18.43 (5, 583)	3.51 (5, 583)	1.65 (5, 583)	1.69 (5, 583)	0.84 (5, 583)	1.75 (5, 583)	0.48 (5, 583)	0.54 (5, 583)
P > *F*	0.7558	0.47	**<0.0001***	**0.0039***	0.145	0.1346	0.5237	0.1224	0.7916	0.7475

[x] Florunner and Georgia Green are TSWV susceptible cultivars and Georgia-06G, Georgia-12Y, Georgia-16HO, and Tifguard are TSWV resistant cultivars; [y] total thrips number on peanut plants counted at 3 to 30 days after thrips release. Means with their standard errors in a column followed by the same letter are not significantly different from each other; [z] F values with degrees of freedom in the parentheses; $p < 0.05$ notated with "*" indicates a significant cultivar effect.
